# Effects of the Quenching Rate on the Microstructure, Mechanical Properties and Paint Bake-Hardening Response of Al–Mg–Si Automotive Sheets

**DOI:** 10.3390/ma12213587

**Published:** 2019-10-31

**Authors:** Guanjun Gao, Yong Li, Zhaodong Wang, Hongshuang Di, Jiadong Li, Guangming Xu

**Affiliations:** State Key Laboratory of Rolling and Automation, Northeastern University, Shenyang 110819, China; wwwgaoguanjun@126.com (G.G.); zhaodongwang@263.net (Z.W.); dhshuang@mail.neu.edu.cn (H.D.); lijd@ral.nue.edu.cn (J.L.); xu_gm@epm.neu.edu.cn (G.X.)

**Keywords:** Al–Mg–Si alloy, quenching rate, microstructures, mechanical properties, paint bake-hardening, precipitates

## Abstract

The quenching rate of Al–Mg–Si alloys during solution treatment is an important parameter for the automotive industry. In this work, the effect of the different quenching rates on the microstructures, mechanical properties, and paint bake-hardening response of Al–Mg–Si sheets was studied. Large dimples form on the fracture surface of a sample at a quenching rate of 0.01 °C/s. When the quenching rate increased to 58.9 °C/s, the dimples became smaller. The recrystallized grains and textures were slightly affected by quenching rates beyond 1.9 °C/s. Thus, higher *r* values of the samples were achieved with slower quenching rates. Furthermore, only the Al(FeMn)SiCr insoluble phases were observed in samples with a rapid quenching rate. Sufficient solute atoms and vacancies resulted in the improvement of the precipitation kinetics and paint bake-hardening capacity for Al–Mg–Si sheets at rapid rates. With a decrease in the quenching rate, the formation of the rod-like coarse β′ phases consumed many solute atoms and vacancies, leading to the deterioration of the paint bake-hardening capacity. This study provides a critical reference on quenching rates for industrial practices, so that good mechanical properties can be achieved using precision control of the quenching process.

## 1. Introduction

In recent years, Al–Mg–Si alloys have become the preferred material for use as automotive outer body sheets due to their high strength-to-weight ratio, good formability, high corrosion resistance, and attractive hardening potential after artificial aging [1,2,3,4]. For heat-treatable Al–Mg–Si alloys, precipitation hardening is the main strengthening mechanism utilized in processing. The precipitation sequence of Al–Mg–Si alloys is as follows [5,6,7,8]: SSSS → clusters/GP zones → pre-β″ → β″ → β′ → β, Si, where SSSS represents a supersaturated solid solution. The main strengthening precipitates are highly coherent GP zones with a large amount of needle-shaped β″ phase after the paint bake-hardening treatment (generally at 185 °C for 20–30 min) [5,9]. At peak aging period, the coarsened β″ phase is semicoherent with the matrix [8]. The post β′ phases are semicoherent rods and produce coarser precipitate microstructures with a lower strength contribution [10,11]. The equilibrium β phase, which is not coherent with the matrix, forms as plates with dimensions of several micrometers composed of Mg_2_Si [12]. The abovementioned precipitation structures and phases are closely related to both the alloy composition and the heat treatment process.

The heat treatment of Al–Mg–Si alloys involves solution annealing, quenching, and aging. The solute atoms redissolve and remain in the SSSS form during solution annealing and quenching. Then, SSSS transforms into strengthening phases after aging [13]. The paint bake-hardening response is obviously affected by the quenching rate. Therefore, the quenching rate is an important parameter during the heat treatment process of automotive sheets. In order to achieve an improved paint bake-hardening response, sufficient supersaturated solute atoms and vacancies are necessary after quenching. Obviously, a faster than critical quenching rate effectively suppresses precipitation during quenching. The alloy composition also affects the microstructure and properties at the same quenching rate. Excessive Si promotes high strength and high conductivity, while excessive Mg plays a negative role in the strength and the conductivity [14]. With a large Mg/Si ratio, the alloy possesses a negative natural aging effect on the maximum hardness during artificial aging, while the alloy with a small Mg/Si ratio shows a negligible negative NA effect [15]. Dispersoids containing Mn and/or Cr have been commonly applied for grain size control during homogenization or annealing treatment. The hardness can increase by inducing Mn and/or Cr dispersoids formed during solution treatment [16,17]. 

Fan et al. [18] found that the rapidly quenched alloy showed a better combination of properties compared to the slowly quenched material in both naturally and artificially aged tempers. Coarse Si and Mg_2_Si precipitates formed during slow quenching affected fracture behavior. Using a series of AlMgSi(Cu) alloys quenched in various fluids, Garric et al. [19] studied the impact of the quenching rate on the microstructure and mechanical properties obtained after aging. The results showed that the usual 10 °C/s could be refined depending on the properties of interest given the applications. However, only three or four quenching rates were studied in their work, and the effect of higher or lower quenching rate has yet to be fully explored. Here, 10 different cooling rates were used during quenching. The main aims were to optimize the quenching rate parameters and to investigate its effects on the microstructures, mechanical properties, and paint bake-hardening response of Al–Mg–Si automotive sheets.

## 2. Materials and Methods

In this study, a commercial cold-rolled AA6016 alloy sheet with a composition of Al—1.10%, Si—0.55%, Mg—0.14%, and Fe—0.10% Cr (wt.%) was provided by MINGTAI Aluminum (Henan, China). Individual sheets, with dimensions of 200 mm × 200 mm × 1 mm, were solution heat-treated at 560 °C for 5 min in an air circulation furnace. After heat preservation, the sheets were directly quenched at 11 different quenching rates to room temperature (RT). The different quenching rates from 58.9 °C/s to 0.01 °C/s were measured using three thermocouples placed on the sheets. The highest quenching rate (58.9 °C/s) was achieved by water cooling, and the other rates were achieved by controlling the temperature and pressure by cooling air. The lowest cooling rate (0.01 °C/s) was achieved by cooling with a 560 °C air furnace. The temperature for each quenching rate was measured from 560 °C to 230 °C, because the range of 230–560 °C was the quenching-sensitive region for this AA6016 alloy.

**Figure 1:**
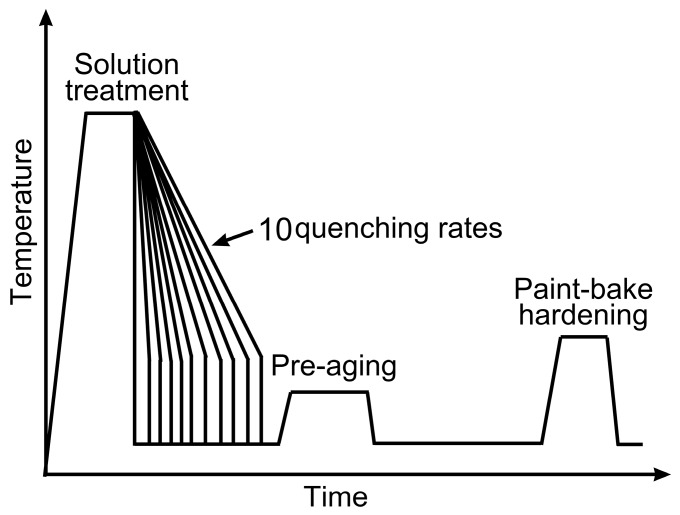
Representation of the heat treatment procedure.

**Figure 2:**
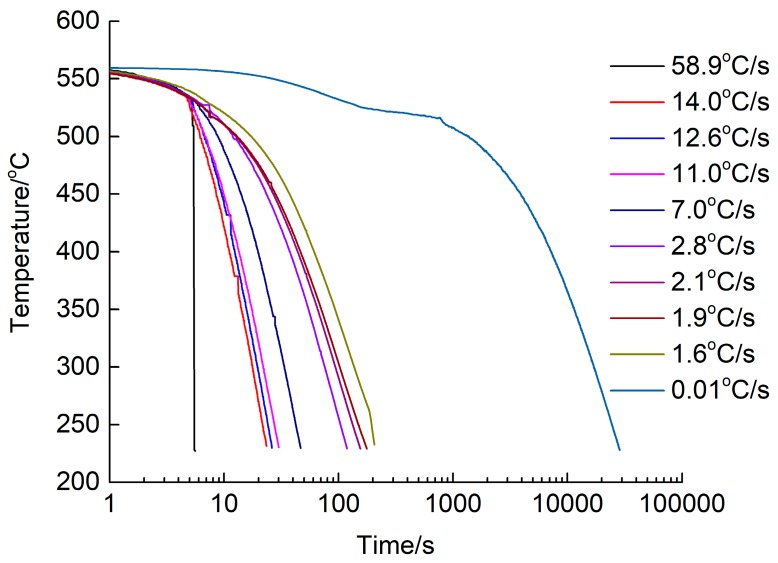
Ten cooling curves of different quenching rates from 58.9 °C/s to 0.01 °C/s.

The as-quenched sheets were immediately pre-aged at 80 °C for 5 h. Subsequently, all sheets were subjected to paint bake-hardening at 185 °C after storage at RT for 168 h. One set of the as-quenched sheets was isothermally heated at 400 °C for 2 h as a control. A schematic representation of the heat treatment procedure is presented in Figure 1, and the measured cooling curves for the as-prepared sheets are presented in Figure 2.

After pre-aging and paint bake-hardening, the sheets were analyzed using the Vickers hardness and tensile tests. The Vickers hardness test was performed using a KB3000BVRZ-SA Vickers hardness tester (Leica Microsystems Company, Germany) with a load of 49 N and a dwell time of 10 s. In order to reduce the testing error, seven indentations were measured, and final values were obtained after removal of maximum and minimum values. The tensile test was performed in accordance with the China Standard (GB/T 228−2002) using an INSTRON-4206 electronic universal tester (Instron Corporation, USA) at a load speed of 3 mm/min. The properties of sheets in three directions of 0°, 45°, and 90° with respect to the rolling direction were tested before and after paint bake-hardening.

The microstructure of the sheets was characterized using an Imager M2m ZEISS optical microscope (ZEISS Company, Germany). The samples were first mechanically ground, followed by electro-polishing in 10 vol % perchloric acid in an alcohol solution at 25 V for 30 s. The surface and fracture morphology were then observed using a ZEISS ULTRA 55 field emission scanning electron microscope (SEM) (ZEISS Company, Germany) at a 15 kV operating voltage, equipped with an X-ray energy dispersive spectrometer (EDS) system. The recrystallization texture of the samples after solution treatment was characterized by electron backscattered diffraction (EBSD) analysis on a ZEISS ULTRA 55 SEM. The step size of the EBSD analysis was set to 2.5 μm. EBSD samples were prepared by electrolytic polishing using a solution of 10% perchloric acid and 90% ethanol at 35 V and 20–40 s.

The size and distribution of particles and precipitates in the samples were investigated using a Tecnai G^2^ F20 transmission electron microscope (TEM) (FEI Company, USA) at a 200 kV operating voltage. The TEM samples were first mechanically ground to approximately 70 μm and then electrolytically polished using a Struers TenuPol-5 jet-polisher (Struers Company, USA) at an operating voltage of 18 V, in an electrolyte solution of 30% by vol nitric acid in methanol (stored between −25 °C and −30 °C). All TEM images were obtained along the <001> Al zone axis.

Differential scanning calorimetry (DSC) samples with a thickness of 0.5 mm, a diameter of 5 mm, and a weight of 25 mg were cleaned using an ultrasonic cleaner. They were then tested using a Q100 model DSC unit (TA Instruments, USA) under an argon atmosphere, and the test temperature ranged from 30 °C to 400 °C with a heating rate of 10 °C/min. Pure aluminum samples were used as reference materials, and the baseline measurements were performed measuring pure aluminum samples.

## 3. Results

### 3.1. Microstructures

**Figure 3:**
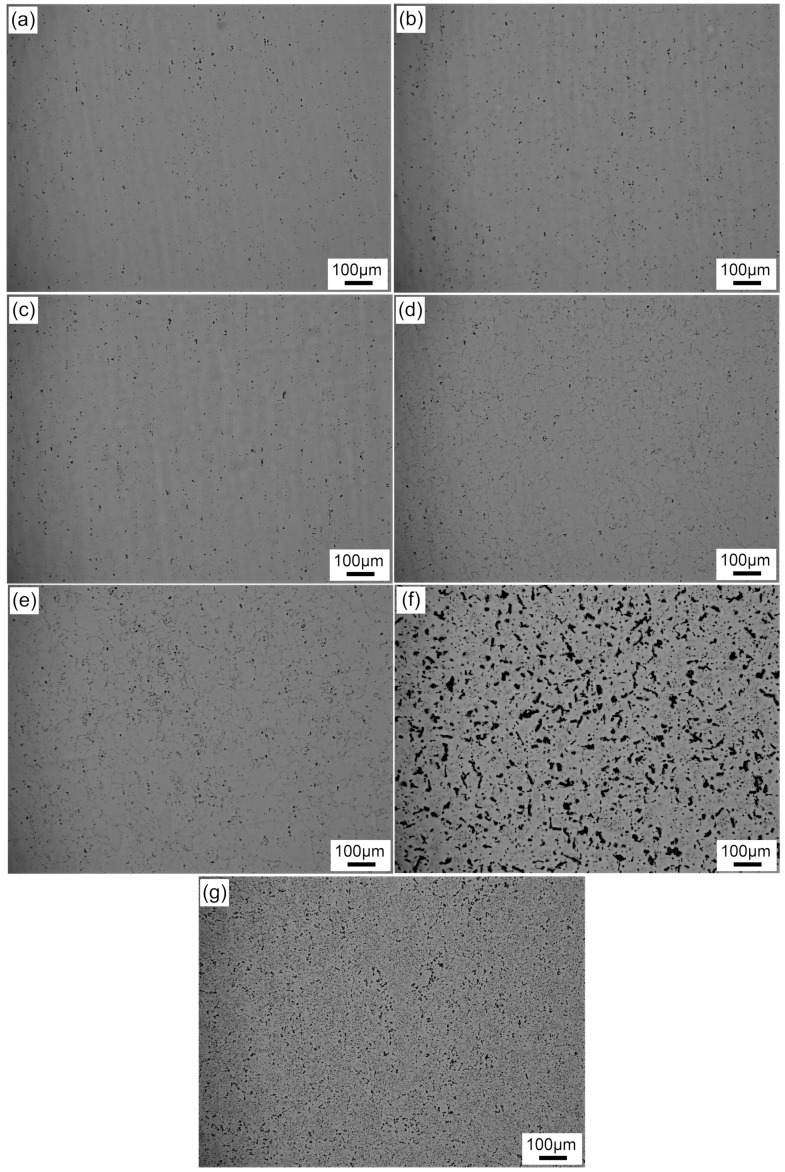
Microstructures of samples with the different quenching rates or annealing treatment at 400 °C for 2 h after pre-aging treatment: (**a**) 58.9 °C/s, (**b**) 14.0 °C/s, (**c**) 7.0 °C/s, (**d**) 2.8 °C/s, (**e**) 1.6 °C/s, (**f**) 0.01 °C/s, and (**g**) annealing at 400 °C for 2 h after water quenching.

Figure 3 shows the microstructures of samples with different quenching rates or annealing treatment at 400 °C for 2 h. No particles were present in the matrix of the sample with a quenching rate of 58.9 °C/s (the black dots in Figure 3a,b were impurities after electro-polishing). With a decrease in the quenching rate, an increasing number of particles were observed in the samples. In particular, the enrichment of the particles was more obvious at the grain boundaries, as shown in Figure 3d,e. Coarse irregular particles formed both in the grains and at grain boundaries when a sample was cooled at the lowest quenching rate (0.01 °C/s). These coarse particles were larger than 20 μm. Meanwhile, some particles below the size of 10 μm were scattered around the coarser ones (Figure 3f). For a sample with annealing treatment at 400 °C for 2 h after water quenching, no coarse particles larger than 20 μm were observed; only a high density of micron particles uniformly distributed in the matrix, as shown in Figure 3g.

**Figure 4:**
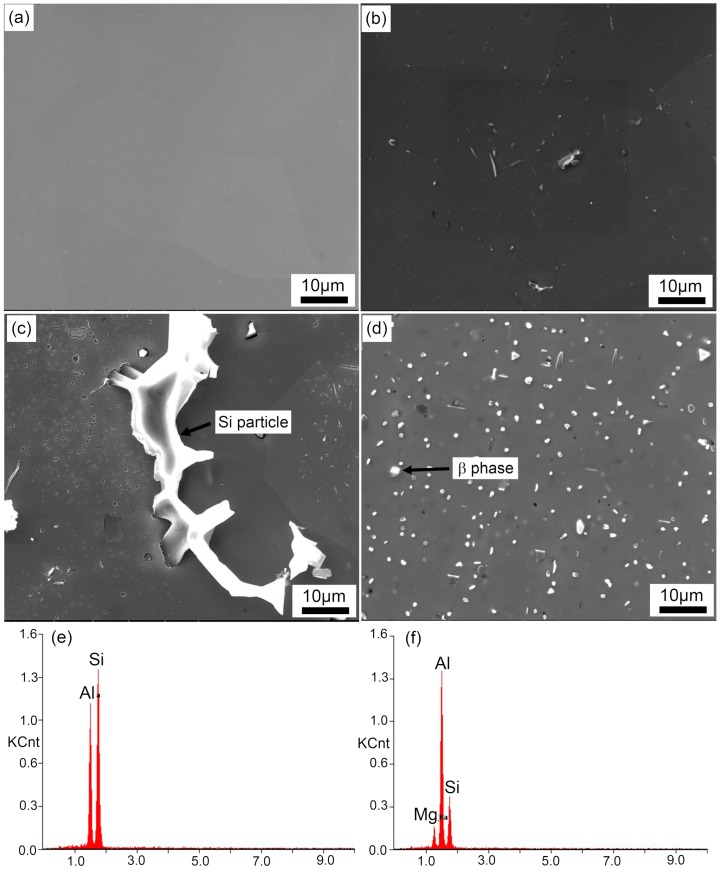
Scanning electron microscope (SEM) micrographs and energy dispersive spectrometer (EDS) analysis of particles distributed in samples with different quenching rates after pre-aging treatment: (**a**) 58.9 °C/s, (**b**) 2.8 °C/s, (**c**) 0.01 °C/s, (**d**) annealing treatment, (**e**) EDS spectra of Si phase, and (**f**) EDS spectra of β phase.

Figure 4 shows SEM micrographs of the particles distributed in the matrix. We found that the size and density of particles increased with decreasing quenching rates. A closer look at the micron particles indicated that these particles were square, spherical, or rod-shaped. According to our EDS analysis (Figure 4f,g), the coarse irregular particles larger than 20 μm were Si phase, while some square particles were β phase (arrows in Figure 4). Thus, we found that an equilibrium β phase was obtained by the annealing treatment method we had adopted here.

**Figure 5:**
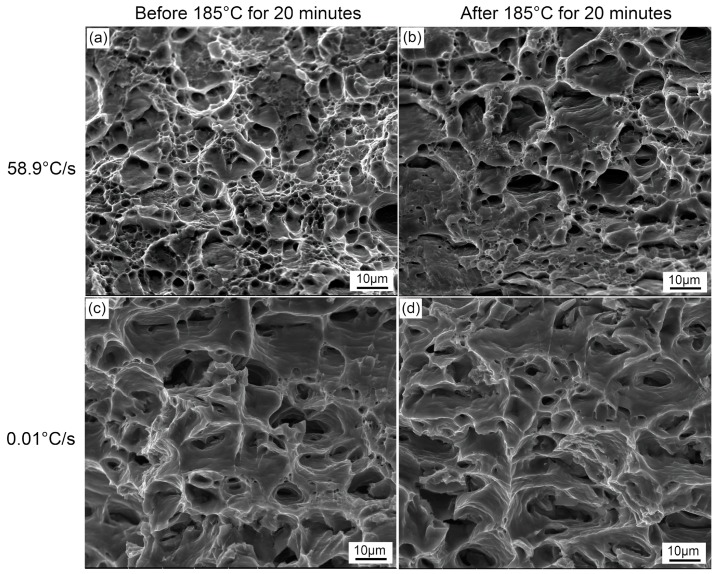
Fracture morphology of samples with different quenching rates before and after paint bake-hardening at 185 °C for 20 min: (**a**) and (**c**), 58.9 °C/s; (**b**) and (**d**), 0.01 °C/s.

Figure 5 shows the fracture morphology of samples at different quenching rates before and after paint bake-hardening at 185 °C for 20 min. A large number of small and deep dimples were uniformly distributed in the 58.9 °C/s quenching rate sample before paint bake-hardening. Conversely, the size and density of the dimples became larger and lower with a decrease in the quenching rate. After paint bake-hardening, a sharp decline in the count occurred for the fast-quenched sample. Some areas of the fracture in this sample presented no dimples, and only river-like streaks were observed. Meanwhile, in the case of the sample with a quenching rate of 0.01 °C/s, the morphology of the fracture changed little. This result indicated that paint bake-hardening had no effect on the morphology of fractures in samples with small quench rates.

### 3.2. Mechanical Property Characterization

**Figure 6:**
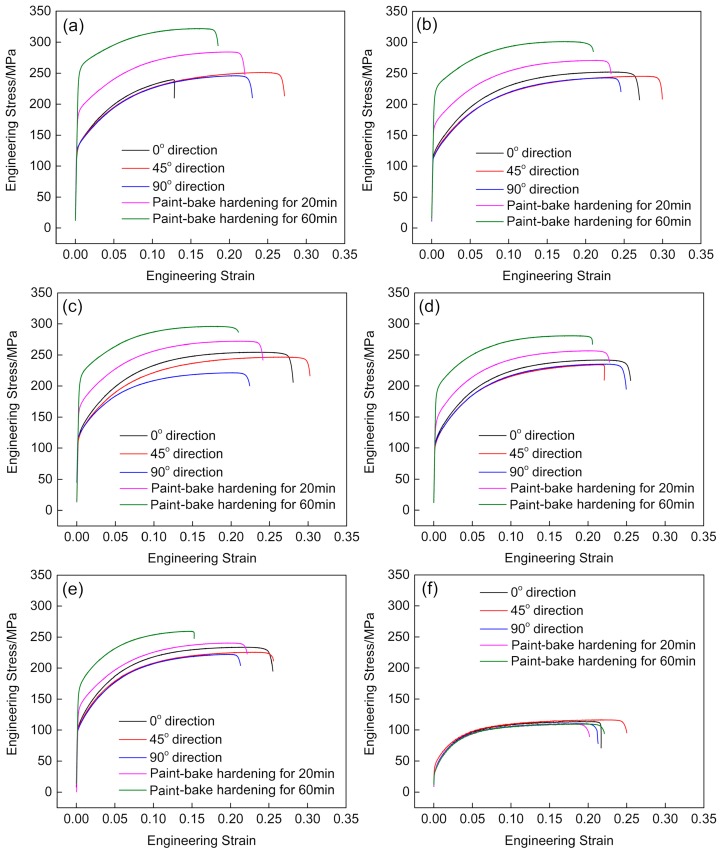
Engineering stress–strain curves of samples with different quenching rates after pre-aging treatment: (**a**) 58.9 °C/s, (**b**) 14.0 °C/s, (**c**) 7.0 °C/s, (**d**) 2.8 °C/s, (**e**) 1.6 °C/s, and (**f**) 0.01 °C/s.

Figure 6 shows the engineering stress–strain curves of samples with different quenching rates before and after paint bake-hardening at 185 °C for 20 min and 60 min. These results revealed that the yield stress of a sample with a quenching rate of 58.9 °C/s before paint bake-hardening was ~130 MPa. Additionally, the yield stress of samples decreased with decreasing quenching rate. When the sample was cooled at a quenching rate of 0.01 °C/s, the yield stress decreased to ~40 MPa. After paint bake-hardening, the strength of the sample increased significantly, and a decrease in elongation occurred. However, the paint bake-hardening treatment had no effect on the strength and elongation of the sample when the quenching rate was decreased to 0.01 °C/s.

**Figure 7:**
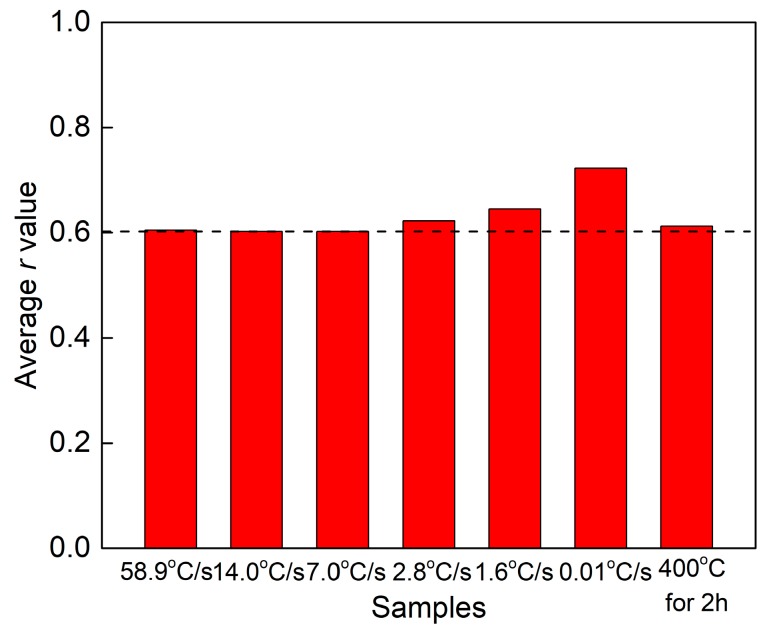
Average *r* value of samples in the T4P state.

In general, the standard for automobile sheets of aluminum requires the *r* value (10% deformation) to be higher than 0.6 [20]. The average *r* value was calculated using

(1) $$\bar{r}=\frac{r_{0^{{}^{\circ}}}+2r_{45^{{}^{\circ}}}+r_{90^{{}^{\circ}}}}{4}$$

where $\;r_{0^{{}^{\circ}}}$, $r_{45^{{}^{\circ}}}$, and $r_{90^{{}^{\circ}}}$ are the *r* values in three different directions [21]. Figure 7 shows the average *r* value of samples with the T4P state (a state of being placed at RT after pre-aging). After pre-aging at 80 °C for 5 h, the average *r* values of samples with different quenching rates were all higher than 0.6, which suggested that a good deep drawability had developed through these treatments. The *r* value remained a constant value of ~0.6 with a quenching rate higher than 7.0 °C/s and heat treatment at 400 °C for 2 h, and this value increased with decreasing quenching rate. The *r* value eventually reached a value of ~0.7 when the quenching rate was dropped to 0.01 °C/s.

**Figure 8:**
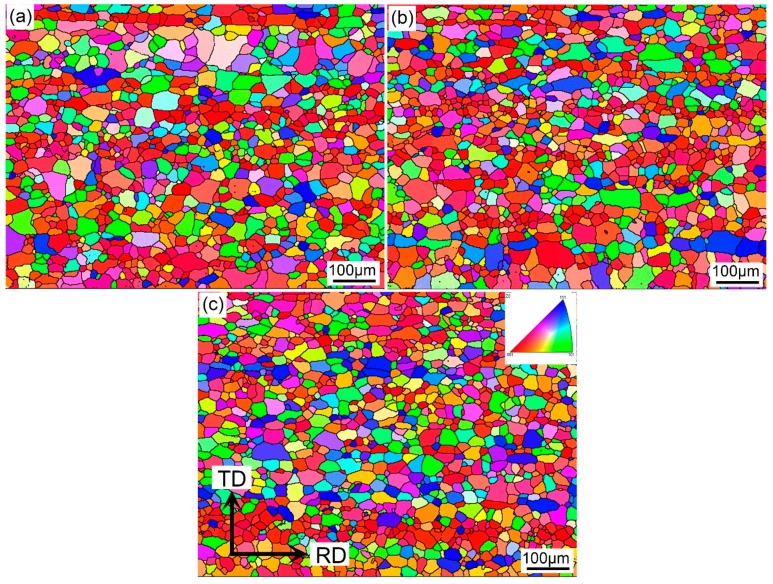
Inverse pole figure (IPF) maps showing the recrystallized grains and texture of the samples with different quenching rates after solution treatment: (**a**) 58.9 °C/s, (**b**) 7.0 °C/s, and (**c**) 1.9 °C/s.

In order to study the average *r* value of these samples, texture analysis was performed on EBSD maps with a scanning size of 2.5 μm. More than 500 grains were included for statistically validity. Figure 8 shows inverse pole figure (IPF) maps displaying the recrystallized grains and texture of samples with different quenching rates after solution treatment. The grains have been completely recrystallized with a solution treatment at 560 °C for 5 min. The average grain size calculated by the linear intercept method was ~22.1, 21.6, and 22.4 μm for the samples with quenching rates of 58.9 °C/s, 7.0 °C/s, and 1.9 °C/s, respectively.

**Figure 9:**
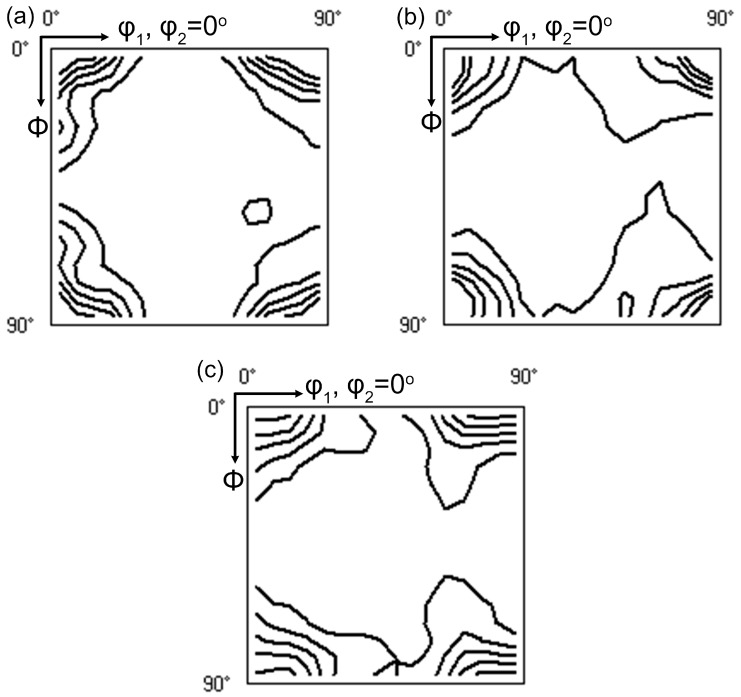
Texture of solutionized samples with different quenching rates: (**a**) 58.9 °C/s, (**b**) 7.0 °C/s, and (**c**) 1.9 °C/s.

Figure 9 shows the final texture of solutionized samples with different quenching rates. We found that their texture components were quite similar. In samples with quenching rates of 58.9 °C/s, 7.0 °C/s, and 1.9 °C/s, the most obvious texture was a recrystallized Cube {001} <100> orientation, which had the highest volume fraction and intensity. In addition, all the samples included Cube_ND_ {001} <310> and P {011} <122> textures that were beneficial to the as-developed deep drawability [22]. The volume fraction and intensity of the texture components changed slightly with the decrease in quenching rate.

### 3.3. Paint Bake-Hardening

**Figure 10:**
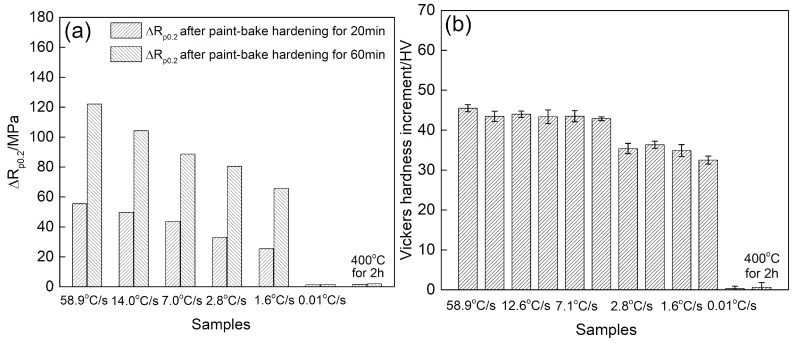
Paint bake-hardening response of samples with different quenching rates: (**a**) Yield stress increment after paint bake-hardening at 185 °C for 20 min and 60 min, and (**b**) Vickers hardness increment after paint bake-hardening at 185 °C for 4 h.

The capacity for paint bake-hardening is an important performance index of automotive body sheets [1,5,23]. After paint bake-hardening treatment, the strength of an alloy sheet is improved to meet the requirements for concave resistance. Figure 10 shows the paint bake-hardening response of samples with different quenching rates. The yield stress of a sample at a quenching rate of 58.9 °C/s increased to 55 MPa and 122 MPa after paint bake-hardening at 185 °C for 20 min and 60 min, respectively. This demonstrated a good capacity for a rapid aging response. However, the strength increment decreased with a decrease in the quenching rate after the paint bake-hardening treatment. When the quenching rate dropped to 0.01 °C/s, no increase in the yield stress occurred (Figure 10a). The peak-aged Vickers hardness values of the samples are presented in Figure 10b. The hardness increment, as well as the yield stress, decreased with decreasing quenching rate. Moreover, the peak-aged hardness did not seriously decrease with a decrease in the quenching rate from 58.9 °C/s to 7.0 °C/s. In addition, no increase in the hardness of a sample with a quenching rate of 0.01 °C/s occurred, even after employing a paint bake-hardening treatment at 185 °C for 4 h. The sample heat treated at 400 °C for 2 h also showed a poor hardening response. As a result, the sample with an extremely slow quenching rate had lost its age-hardening capacity.

### 3.4. Precipitation Observation

**Figure 11:**
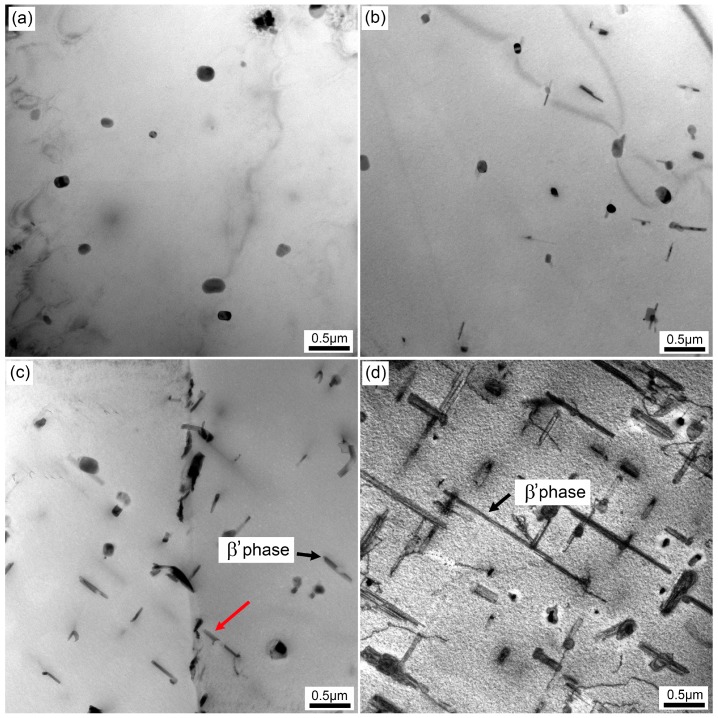
TEM bright-field images of samples with different quenching rates after paint bake-hardening at 185 °C for 4 h: (**a**) 58.9 °C/s, (**b**) 7.0 °C/s, (**c**) 2.8 °C/s, and (**d**) 1.6 °C/s.

To further investigate the paint bake-hardening response of the samples with different quenching rates, the precipitates in the matrix were observed using TEM. Figure 11 shows the TEM bright-field images of the samples with different quenching rates after paint bake-hardening at 185 °C for 4 h. In the sample with the quenching rate of 58.9 °C/s, only spherical particles of 100–200 nm size were observed, and uniformly distributed in the matrix, while in the sample with the quenching rate of 7.0 °C/s, a small amount of rod-like precipitates appeared. The length of the precipitates was ~500 nm, and the width was less than 50 nm. Obviously, a certain orientation relationship existed between the rod-like precipitates and the matrix. With the decrease of quenching rate, the density and size of these rod-like precipitates increased significantly. These rod-like precipitates co-existed with the spherical particles in the matrix. When the quenching rate dropped to 1.6 °C/s, these rod-like phases were seriously coarsened and lengthened. The width of the phases was ~200 nm, and the length reached ~3000 nm.

**Figure 12:**
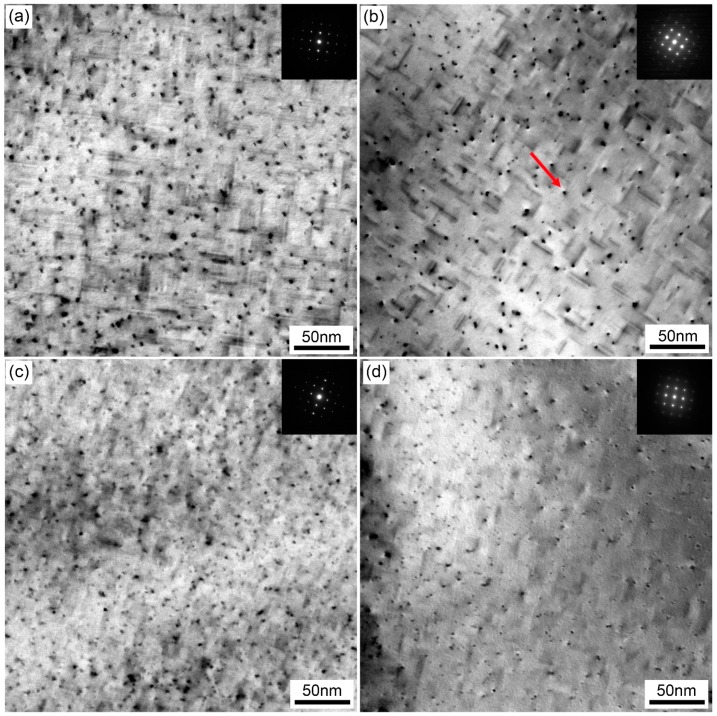
Enlarged TEM bright-field images of samples with different quenching rates after paint bake-hardening at 185 °C for 4 h: (**a**) 58.9 °C/s, (**b**) 7.0 °C/s, (**c**) 2.8 °C/s, and (**d**) 1.6 °C/s.

Figure 12 shows enlarged TEM bright-field images of samples with different quenching rates. A large amount of fine dot-like and needle-like precipitates was observed in the matrix. Previous studies [24] have indicated that these dot-like precipitates were the needle-like ones viewed in another direction. In samples with different quenching rates, the density and size of the precipitates in the matrix significantly dropped (from ~40 nm to ~15 nm) when the quenching rate decreased from 58.9 °C/s to 7.0 °C/s. As the quenching rate continued to drop to 2.8 °C/s, the size of the needle-like precipitates decreased in width. In a sample with a quenching rate of 1.6 °C/s, the density of the precipitates further decreased in the matrix.

**Figure 13:**
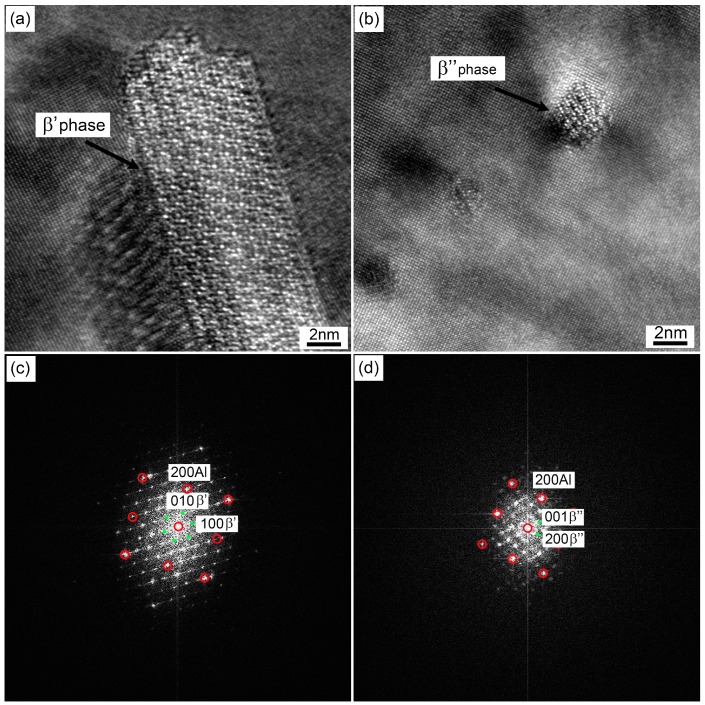
(**a**,**b**), HRTEM images of the large rod-like and fine needle-like precipitates in Figure 11 and Figure 12 (red arrows marked); and (**c**,**d**), the corresponding FFT (Fast Fourier Transform) patterns from samples in (**a**,**b**).

In order to further study the effect of these precipitates in samples with different quenching rates on the paint bake-hardening response, HRTEM analysis and the corresponding FFT (Fast Fourier Transform) patterns based on the large rod-like and fine needle-like precipitates in Figure 11 and Figure 12 were analyzed, as shown in Figure 13. According to the corresponding FFT patterns, the rod-shaped precipitate was identified as a late phase β′. The orientation relationships between these rod-like precipitates and the matrix were (001)//(001)_Al_, [010]//[100]_Al_, and [100]//[$\bar{3}$50]_Al_. This observation was consistent with previous studies by Vissers et al. [11]. Additionally, the orientation relationships between the fine needle-like precipitates and the matrix were (010)//(00$\bar{1}$)_Al_, [001]//[130]_Al_, and [100]//[3$\bar{2}$0]_Al_. Based on the HRTEM and corresponding FFT patterns, the precipitates observed were identified as being β″ phase, which was the main hardening precipitate for a Al–Mg–Si alloy system [8,25,26].

## 4. Discussion

It is an often noted fact that dispersed particles and strengthening precipitates (β″ and β′ precipitate) are common in Al–Mg–Si alloys. The size and density of these precipitates are greatly influenced by quenching rates [27,28]. These dispersed particles change slightly during subsequent heat treatments [29]. When a sample was cooled to RT with a very slow quenching rate, large-sized Si particles formed in the matrix. During the fracture process of this alloy, most cracks germinated in the vicinity of these coarse particles and became favorable sites for crack nucleation [30], resulting in the large dimples in Figure 5b,d. However, the strength of the sample was very low at a quenching rate of 0.01 °C/s, regardless of the paint bake-hardening treatment (Figure 6). The improvement in plasticity caused by this decrease in strength was the main reason for the formation of the large dimples. In contrast, no coarse particles formed in the matrix of a sample with a quenching rate of 58.9 °C/s after pre-aging treatment, and the strength was relatively higher. Thus, the dimple size of the fracture surface decreased. After paint bake-hardening treatment, precipitates rapidly grew in a one-dimensional direction, and the strength of the sample increased, while the plasticity decreased [5,31]. This resulted in a further decrease in the size of dimples, and in some areas, no dimples existed.

During solution treatment, the rolled microstructure transformed into recrystallized grains, and the soluble phase redissolved into the matrix. This recrystallization was prior to the redissolution of the soluble phase. If the alloy was kept at a high temperature for a long time, grain boundary migration occurred. With the grain boundary migration, the larger grains swallowed up adjacent smaller grains, resulting in an increase in the average grain size [32,33]. In this study, when the sample was cooled to RT with a very slow quenching rate of 1.9 °C/s, the driving force provided by external energy at high temperature was not enough to cause grain boundary migration or abnormal grain growth. The grain size of a sample with a quenching rate of 1.9 °C/s was similar to that of 58.9 °C/s. Furthermore, the volume fraction and intensity of main recrystallization texture changed slightly with decreasing quenching rate (Figure 8 and Figure 9), while the average *r* value increased with decreasing quenching rate. Generally, the *r* value of alloy sheets is closely related to recrystallized grain orientations [21,34,35]. Retaining a rolling texture such as S {123} <634> or B {011} <211> leads to a higher *r* value. In contrast, a recrystallized cube texture {001} <100> results in a lower *r* value. According to our experimental results, although the recrystallized textures of samples with the different quenching rates were similar, the *r* values increased with decreasing quenching rate. This may have been due to the coarse Si phase formed with this small quenching rate, which hindered the strain in the thickness direction more effectively than in the width direction. Therefore, the coarse Si phase was the main contributor to the increase in the *r* value.

It has been reported previously that the precipitation sequence for Al–Mg–Si alloys is SSSS → clusters/GP zones → pre-β″ → β″ → β′ → β, Si. In this study, no coarse rod-like β′ precipitates formed in the matrix, and only spherical particles were observed in a sample with a quenching rate of 58.9 °C/s (Figure 11a). These spherical particles were confirmed to be an Al(FeMn)SiCr insoluble phase in previous research [13]. Although the samples were treated by paint bake-hardening at 185 °C for 4 h, only nanometer-scale needle-like β″ precipitates formed, rather than rod-like β′ precipitates. With a decrease in the quenching rate, the residence time of samples at higher temperature was prolonged. Sufficient energy promoted the rapid nucleation and growth of precipitates, leading to the formation of micron-sized β′ precipitates. As the quenching rate continued to decrease, the density and size of the β′ precipitates further increased (Figure 11d). When a sample was cooled with an extremely small quenching rate (0.01 °C/s), all solute atoms were separated from the matrix. A large amount of coarse Si phase accompanied by β′ phase formed, which was observed by optical microscopy (Figure 3e). The consumption of supersaturated solute atoms and vacancies led to the decrease of cluster density during pre-aging treatment, which decreased the strength of the alloy [36].

**Figure 14:**
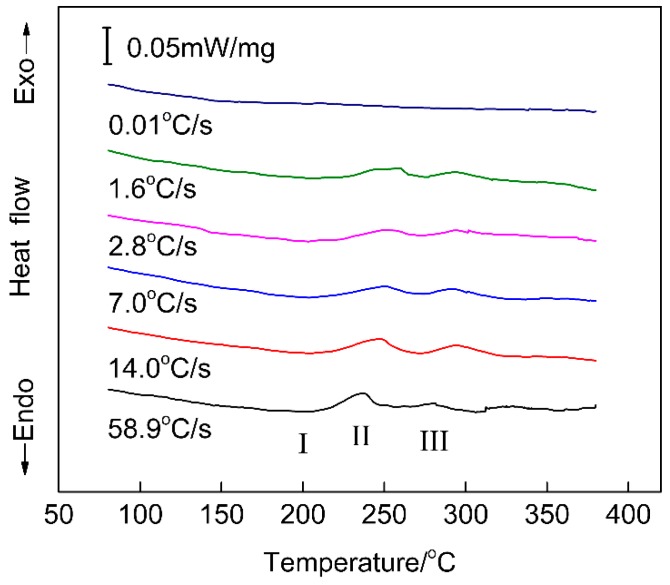
Differential scanning calorimetry (DSC) curves for samples with different quenching rates.

In the practice of automotive manufacturing, the general paint bake-hardening treatment is to hold an alloy at 185 °C for 20–30 min. During a paint bake-hardening treatment, precipitates rapidly grow in a one-dimensional direction, and an elastic distortion region forms around them. This effectively hinders the dislocation movement, resulting in the occurrence of a rapid aging response [5,8]. When the time of paint bake-hardening treatment at 185 °C is extended to 4–10 h, most precipitates transform into β″ phase. In the peak-aged state, the hardness of the alloy reaches its maximum. In this study, DSC analysis of samples with different quenching rates was performed to investigate this precipitation behavior, as shown in Figure 14. We observed that the temperature of peak II related to β″ phase increased with decreasing quenching rate. For a sample with a quenching rate of 0.01 °C/s, no endothermic or exothermic reaction occurred during DSC heating. Consequently, a higher density of β″ phase formed in the rapidly quenched sample when holding at a temperature for the same amount of time.

As shown in Figure 12, the density and size of the β″ precipitates decreased with quenching rate decrease after a paint bake-hardening treatment at 185 °C for 4 h. For a sample with a quenching rate of 58.9 °C/s, all solute atoms dissolved into the matrix to form SSSS. When a sample was rapidly quenched to RT, there were many supersaturated solute atoms and vacancies in the matrix. During the subsequent paint bake-hardening treatment, sufficient solute atoms and vacancies provided the raw materials and diffusion channels for the formation of β″ strengthening phases. Not only that, with solute atoms and vacancies, more nucleation sites were provided for the formation of β″ precipitates [37,38,39]. The abovementioned factors ensured that the strength and hardness of a sample obviously increased after paint bake-hardening treatment (Figure 10). Additionally, the diffusion distance was short due to sufficient vacancies. The formation of the β″ precipitates needed less energy. Hence, this obviously decreased the precipitation temperature of β″ phase structures (Figure 14).

When the quenching rate of the sample decreased, the hold time at high temperature was prolonged. A large number of solute atoms and vacancies were consumed to form a coarse β′ phase structure (Figure 13). This led to a decrease in the density and size of β″ precipitates after a paint bake-hardening treatment [36,40]. Compared with β′ phase, the strengthening effect of β″ precipitates was more remarkable [5,41]. Therefore, the strength and hardness increment decreased with decreasing quenching rate. Furthermore, the consumption of solute atoms and vacancies increased the diffusion distance, and the nucleation and growth of β″ precipitates needed more energy. Thus, an exothermic peak related to the β″ phase appeared at higher temperatures in the DSC curves (Figure 14). For a sample with an extremely slow quenching rate (0.01 °C/s), the solute atoms and vacancies were almost consumed, leading to no precipitation reaction during the DSC heating process.

## 5. Conclusions

The effects of different quenching rates on the microstructure, mechanical properties, and paint bake-hardening response of Al—1.10% and Si—0.55% Mg automotive sheets were investigated in this study. The conclusions are as follows:

1. For a sample with a quenching rate of 0.01 °C/s, large dimples formed on the fracture surface due to the coarse Si phase and good plasticity. With a quenching rate of 58.9 °C/s, these dimples became smaller. The plasticity of a sample rapidly deteriorated, and the size of dimples further decreased after paint bake-hardening treatment;
2. The recrystallized grains and textures were slightly affected by the quenching rate beyond 1.9 °C/s. While higher *r* values of samples with low quenching rates were achieved, this may have been related to the hindrance of coarse particles due to the strain in thickness direction during the deformation of the sample;
3. For a sample with a rapid quenching rate, only the Al(FeMn)SiCr insoluble phase was observed. With a decrease in the quenching rate, the holding time at higher temperature was prolonged. This led to sufficient energy in the system, which promoted the nucleation and growth of precipitates, leading to the formation of rod-like coarse β′ phase structures;
4. Sufficient solute atoms and vacancies were formed in a sample with a rapid quenching rate, resulting in the improvement of the precipitation kinetics and paint bake-hardening capacity. With a decrease in the quenching rate, the solute atoms and vacancies were consumed. Consequently, the precipitation kinetics of the β″ phase decreased, and the paint bake-hardening capacity deteriorated.
